# Into the limelight: TPA for critically ill Covid‐19 patients

**DOI:** 10.1002/jha2.429

**Published:** 2022-06-08

**Authors:** Eslam Abbas, Ahmed Mahdy, Rawad Tarek, Nawar Jabbour, Huda Al‐Foudri

**Affiliations:** ^1^ Department of OncoCardiology Dar El Salam Cancer Center Cairo Egypt; ^2^ Department of Cardiology Al Adan Hospital Kuwait City Kuwait; ^3^ Department of Intensive Care Al Adan Hospital Kuwait City Kuwait

In response to Cromartie and Prin letter, the authors would like to mention that:

1. In the setting of Covid‐19 patients on ECMO, monitoring of anticoagulation therapy was performed using ACT with a reference range between 180 and 220 s.
2. The alteplase dose was chosen according to a systematic review and meta‐analysis by Zhang et al. [1] that compared the use of low‐dose rt‐PA (0.6 mg/kg, maximum 50 mg or 50 mg infused over 2 h) with standard dose (100 mg infused over 2 h) for the treatment of pulmonary embolism (PE) and concluded that low‐dose rt‐PA had similar efficacy but was safer than standard dose of rt‐PA. Also, the ESC guidelines [2] of treatment of PE approved the low‐dose protocol with rapid infusion in case of rapid hemodynamic deterioration.
3. The most serious side effect of lysis therapy is bleeding, which can be life threatening with an ominous prognosis [3]. Major bleeding was defined as fatal bleeding or overt bleeding with a drop in hemoglobin level of at least 20 g/L or requiring transfusion of at least two units packed blood cells, or hemorrhage into a critical anatomical site (such as intracranial, intraspinal, intraocular, retroperitoneal, or pericardial) [4, 5]. Minor bleeding was defined as any acute or subacute clinically overt bleeding that did not satisfy the criteria for major bleeding. ADEs, especially ICH, were assessed clinically during daily sedation interruptions. There was no detectable acute hemoglobin drop, and PAUS was used for follow‐up.
4. The main recommendations of our report [6] are the following: (i) bedside echocardiography can be a beneficial tool in the urgent assessment of the right ventricle‐to‐pulmonary vascular coupling in mechanically ventilated Covid‐19 patients and (ii) a low‐dose protocol of lysis therapy may be beneficial in mechanically ventilated, hemodynamically unstable Covid‐19 patients who show echocardiographic criteria of right ventricular strain. The decision of implementing lysis therapy should weigh the current mortality rate of this patient subset in the absence of a specific antiviral treatment, besides the proposed benefits of clinical improvement against the possible bleeding complication of such intervention.
